# Case report: Ultrasound-guided needle knife technique for carpal ligament release in carpal tunnel syndrome treatment

**DOI:** 10.3389/fneur.2023.1291702

**Published:** 2023-11-09

**Authors:** Jianjun Sun, Xiaodi Zou, Qinyun Fu, Jianhua Wu, Shuaishuai Yuan, Ahmad Alhaskawi, Yanzhao Dong, Haiying Zhou, Sahar Ahmed Abdalbary, Hui Lu

**Affiliations:** ^1^Department of Anesthesiology, Pujiang County Hospital of Traditional Chinese Medicine, Jinhua, China; ^2^Second Affiliated Hospital, Zhejiang Chinese Medical University, Hangzhou, Zhejiang, China; ^3^First Affiliated Hospital, School of Medicine, Zhejiang University, Hangzhou, Zhejiang, China; ^4^Nahda University, Beni Suef, Beni Suef, Egypt

**Keywords:** minimally invasive surgery, ultrasound-guided, needle knife technique, carpal tunnel syndrome, carpal tunnel release

## Abstract

Carpal tunnel syndrome (CTS) is a common peripheral neuropathy of the hand, mainly manifesting as sensory disturbances, motor dysfunctions, and pain in the fingers and hand. The pathogenesis of the disease is associated with fibrosis of the transverse carpal ligament in the carpal tunnel, which compresses median nerve. In our case, we demonstrate an ultrasound-guided needle knife technique to treat CTS. We guided the patient to a supine position on the examination table. The skin of the wrist area was sterilized for the procedure. After the skin was dry, we positioned sterile drapes, located the median nerve and compression, and marked the compression point. Local anesthesia was administered. An ultrasound-guided needle knife was inserted. The needle knife was advanced under ultrasound guidance. The carpal ligament was incised. A gradual release of pressure on the median nerve was observed on the ultrasound monitor. After treatment, the patient’s finger sensation and motor function can significantly improve, and pain symptoms are markedly reduced, this case demonstrates that small needle-knife treatment can serve as a safe and effective minimally invasive therapeutic method.

## 1. Introduction

Carpal tunnel syndrome (CTS) is a prevalent and debilitating condition characterized by the compression of the median nerve as it passes through the carpal tunnel at the wrist. CTS (Carpal Tunnel Syndrome) is one of the common peripheral nerve injury disorders (1). This compression results in symptoms such as hand numbness, tingling, and pain, often impairing a patient’s ability to perform daily activities. Diagnosis of CTS typically involves a combination of clinical tests and electrodiagnostic studies, some common clinical tests include Phalen’s test, carpal compression test, Tinel’s test, and Durkan’s compression test (2–6). These tests help assess the sensitivity, specificity, and predictive values for CTS diagnosis. However, relying on a single test may not be sufficient for definitive diagnosis, and a combination of clinical and paraclinical tests may be necessary (3). Nerve conduction studies (NCS) are often used to confirm CTS diagnosis based on the latency, amplitude, distance, and velocity of the median and ulnar nerves (7). Ultrasound has emerged as a noninvasive diagnostic alternative to NCS for CTS (8). It evaluates the median nerve’s cross-sectional area, regional echogenicity, and coexisting pathologies that may increase pressure in the carpal tunnel.

Conservative methods for CTS include non-surgical treatments such as splinting, physiotherapy, corticosteroid, neurodynamic techniques, gabapentin, therapeutic ultrasound, and extracorporeal shockwave therapy (9). However, conservative treatment has been effective for mild and moderate idiopathic (10). Traditional surgical interventions for CTS involve a number of different methods for surgical release, including traditional open dissection, double incision cannula (11), single-small incision release (12), and endoscopic release (13). However, there remain several controversies regarding the advantages and disadvantages of various surgical methods, such as longer recovery times, scarring, and potential complications. (14). In recent years, there has been growing interest in the development of minimally invasive techniques for carpal ligament release, aimed at alleviating the compression on the median nerve. One such technique that has gained attention is the ultrasound-guided needle knife technique. This innovative approach combines the precision of ultrasound imaging with a minimally invasive needle knife to achieve carpal ligament release. By directly visualizing the anatomy and pathology of the carpal tunnel using ultrasound, clinicians can accurately guide the needle knife to the point of ligamentous compression, thus reducing the risk of injury to surrounding structures. Ultrasound guidance can also diagnose other causes of median nerve injury, such as tumors, gout, and more (15–17).

The ultrasound-guided needle knife technique offers several potential advantages over traditional open surgical approaches. First and foremost, it allows for a smaller incision and reduced tissue trauma, leading to quicker postoperative recovery and decreased scarring. Additionally, the real-time visualization provided by ultrasound ensures accurate targeting of the compressed median nerve, minimizing the risk of inadvertent nerve or vessel damage. This technique has the potential to provide patients with a more efficient and less painful treatment option, ultimately improving their quality of life. After the treatment of ultrasound-guided needle knife technique, we can judge the effectiveness and safety of the treatment by ultrasonography, which can assess median nerve and carpal tunnel dimensions, median nerve position within the tunnel, and flexor retinacular bowing and thickness (18).

In this protocol, we present a detailed description of the ultrasound-guided needle knife technique for carpal ligament release in the treatment of carpal tunnel syndrome. We outline the steps involved in the procedure, from preoperative preparation to postoperative care. Furthermore, we discuss the potential benefits and limitations of this technique and its significance in the context of existing treatment methods. By offering a less invasive alternative, we aim to contribute to the advancement of effective and patient-friendly approaches for managing carpal tunnel syndrome. And we provide a comprehensive guide to implementing this innovative technique and discuss its potential implications in the field of hand surgery.

## 2. Protocol

### 2.1. Reoperative preparation

#### 2.1.1. Informed consent

Before initiating the procedure, ensure thorough communication with the patient regarding the ultrasound-guided needle knife technique. Explain the purpose of the procedure, the potential benefits, risks (puncture site infection, bleeding at the puncture site, median nerve injury et al.), and alternatives. Obtain written informed consent from the patient, addressing any concerns or questions they may have.

#### 2.1.2. Patient positioning

In an operating room, gently guide the patient to a comfortable supine position on the examination table. Instruct the patient to extend the affected hand with the palm facing upward. Encourage relaxation to minimize muscle tension during the procedure.

#### 2.1.3. Skin preparation and sterilization

Prepare the skin for the procedure by cleaning the wrist area using an antiseptic solution. Begin by cleaning the entire wrist and hand, including the palm and fingers. Use a circular motion while cleaning, moving from the center outward to create a sterile field.

#### 2.1.4. Application of sterile drape

Once the skin is clean and dry, carefully position a sterile drape over the wrist area. Ensure that the drape covers the entire wrist and hand, leaving only the target area exposed for the procedure. Maintain the sterility of the draped area throughout the procedure.

#### 2.1.5. Anesthesia discussion

Engage in a discussion with the patient about local anesthesia administration. Address the type of anesthesia to be used, the sensation they might experience, and its purpose in ensuring their comfort during the procedure. Reiterate their ability to communicate any discomfort or sensations during the procedure.

#### 2.1.6. Verification of patient identity

Before proceeding, verify the patient’s identity by confirming their name and date of birth. Cross-reference this information with their medical records and the consent form to ensure accurate patient identification.

#### 2.1.7. Patient comfort and reassurance

Take a moment to ensure the patient’s comfort and address any last-minute concerns they may have. Reiterate the steps of the procedure and emphasize your commitment to their well-being and safety throughout the process. An ECG monitor is connected to the patient.

### 2.2. Ultrasound-guided localization of median nerve

#### 2.2.1. Application of sterile ultrasound gel

Dispense a sufficient amount of sterile ultrasound gel onto the wrist area where the median nerve is expected to be located. The gel enhances acoustic coupling between the skin and the ultrasound transducer, facilitating clear visualization of the underlying structures.

#### 2.2.2. Ultrasound transducer placement

Hold the ultrasound transducer (WISONIC WA55-23020232022-03) with a sterile transducer cover in your dominant hand and gently position it over the wrist area covered with ultrasound gel. Orient the transducer to obtain a longitudinal view of the wrist, aligning it parallel to the forearm. Gradually adjust the transducer’s angle and orientation until you obtain a clear view of the median nerve.

#### 2.2.3. Identification of median nerve and compression point

Slowly slide the ultrasound transducer along the wrist while observing the real-time ultrasound images on the monitor. Identify the median nerve as a hypoechoic structure with internal fascicular patterns. Differentiate the nerve from surrounding tendons and ligaments.

Using the ultrasound imaging, identify the specific point where the median nerve is compressed within the carpal tunnel. This point may exhibit hypoechoic changes or narrowing of the nerve diameter, indicating the site of compression. Measure the diameter of the compressed portion of the median nerve to quantify the degree of compression.

#### 2.2.4. Marking the compression point

With the compression point identified and visualized on the ultrasound monitor, use a sterile marking pen to place a small dot on the patient’s skin directly above the compressed region. This mark will serve as a reference point during the subsequent steps of the procedure.

#### 2.2.5. Documentation and image capture

Capture representative ultrasound images of the median nerve in both its normal and compressed states. These images will serve as visual documentation for patient records and can be referred to during the procedure to ensure accurate needle placement.

### 2.3. Needle knife technique for carpal ligament release

#### 2.3.1. Administration of local anesthesia

Prepare a syringe containing the predetermined volume of local anesthetic (2% lidocaine 5 mL) with a fine needle attached. Administer local anesthesia at the marked site over the compressed median nerve. Insert the needle gently, avoiding excessive pressure. Slowly inject the anesthetic while monitoring the patient’s comfort and response. Wait for a few moments to allow the anesthetic to take effect.

#### 2.3.2. Insertion of ultrasound-guided needle knife

Once adequate anesthesia is achieved, prepare the ultrasound-guided needle knife. Hold the needle knife (HUAYOU small needle-knife 0.6*0.8 mm) in your dominant hand, positioning it at the marked site (0.5 cm above transverse striation of palm wrist) over the compressed median nerve. Align the needle knife(at an insertion angle of 30°) with the orientation observed on the ultrasound monitor.

#### 2.3.3. Needle knife advancement under ultrasound guidance

Begin advancing the needle knife slowly and steadily under continuous ultrasound guidance. As you progress, closely monitor the needle’s trajectory on the ultrasound monitor to ensure accurate positioning in relation to the compressed median nerve. Adjust the angle and depth of insertion as necessary to maintain the needle’s alignment with the target site.

#### 2.3.4. Carpal ligament incision

With the needle knife accurately positioned near the compressed median nerve, initiate the incision of the carpal ligament. Employ controlled and deliberate movements, ensuring the needle knife’s trajectory remains aligned with the nerve. As you incise the ligament, observe for any reduction in tension or pressure on the median nerve, which may be visualized on the ultrasound monitor.

#### 2.3.5. Monitoring patient comfort and anesthesia adjustment

Throughout the procedure, maintain open communication with the patient. Inquire about their comfort level and any sensations they may experience. If the patient expresses discomfort or pain, administer additional local anesthesia as needed. It is essential to prioritize the patient’s well-being and ensure they remain comfortable throughout the intervention.

#### 2.3.6. Continuous ultrasound visualization

During the entire needle knife technique, maintain continuous visualization of the needle’s position and its proximity to the median nerve using the ultrasound monitor. Adjust the needle’s orientation and trajectory as necessary to optimize accuracy and avoid unintended structures.

#### 2.3.7. Gradual release of pressure

As the carpal ligament is incised, you may observe a gradual release of pressure on the compressed median nerve on the ultrasound monitor. This reduction in compression should result in morphological changes of the previously compressed median nerve, which can be visually assessed during the procedure.

### 2.4. Postoperative care and follow-up

#### 2.4.1. Application of sterile dressing

Following the completion of the needle knife technique, ensure hemostasis at the incision site. Gently clean the area with sterile saline solution to remove any residual blood or debris. Apply a sterile dressing over the incision site to protect it from external contaminants and to facilitate wound healing. Ensure that the dressing is secure but not too tight, allowing for adequate circulation.

#### 2.4.2. Wrist elevation and activity restrictions

Instruct the patient to keep the treated wrist elevated for the first 24 to 48 h after the procedure. Elevating the wrist helps minimize swelling and promotes fluid drainage. Advise the patient to avoid engaging in strenuous activities or heavy lifting for several days postoperatively to prevent unnecessary strain on the healing tissues.

#### 2.4.3. Pain medication and antibiotics

Prescribe pain medication as necessary to manage postoperative discomfort. Educate the patient on the proper dosage and administration schedule of the prescribed pain relievers. Additionally, if deemed appropriate, prescribe a short course of antibiotics to minimize the risk of infection at the incision site.

#### 2.4.4. Scheduling a follow-up appointment

During the patient’s discharge, schedule a follow-up appointment for a comprehensive assessment of their recovery progress. This appointment should occur within a suitable timeframe, typically 1 to 2 weeks postoperatively. During the follow-up visit, assess whether the puncture site is infected, pain levels, and overall comfort. Assess the effectiveness of the procedure in alleviating symptoms and improving median nerve function.

#### 2.4.5. Patient education

Provide the patient with detailed postoperative instructions in written form. Include information on wound care, signs of infection, and guidelines for gradual resumption of normal activities. Emphasize the importance of adhering to the prescribed medication regimen and attending the scheduled follow-up appointment.

#### 2.4.6. Monitoring and complications

Educate the patient about potential complications that may arise after the procedure, such as infection, bleeding, or persistent discomfort. Instruct them to promptly seek medical attention if they experience worsening pain, signs of infection (redness, swelling, warmth, pus), or any concerning symptoms.

#### 2.4.7. Rehabilitation and physical therapy

Consider incorporating rehabilitation and physical therapy into the patient’s recovery plan. Collaborate with rehabilitation specialists to design a tailored program that aims to restore wrist strength, mobility, and function. Rehabilitation can help accelerate the patient’s return to regular activities while minimizing the risk of complications.

## 3. Representative results

**Figure 1:**
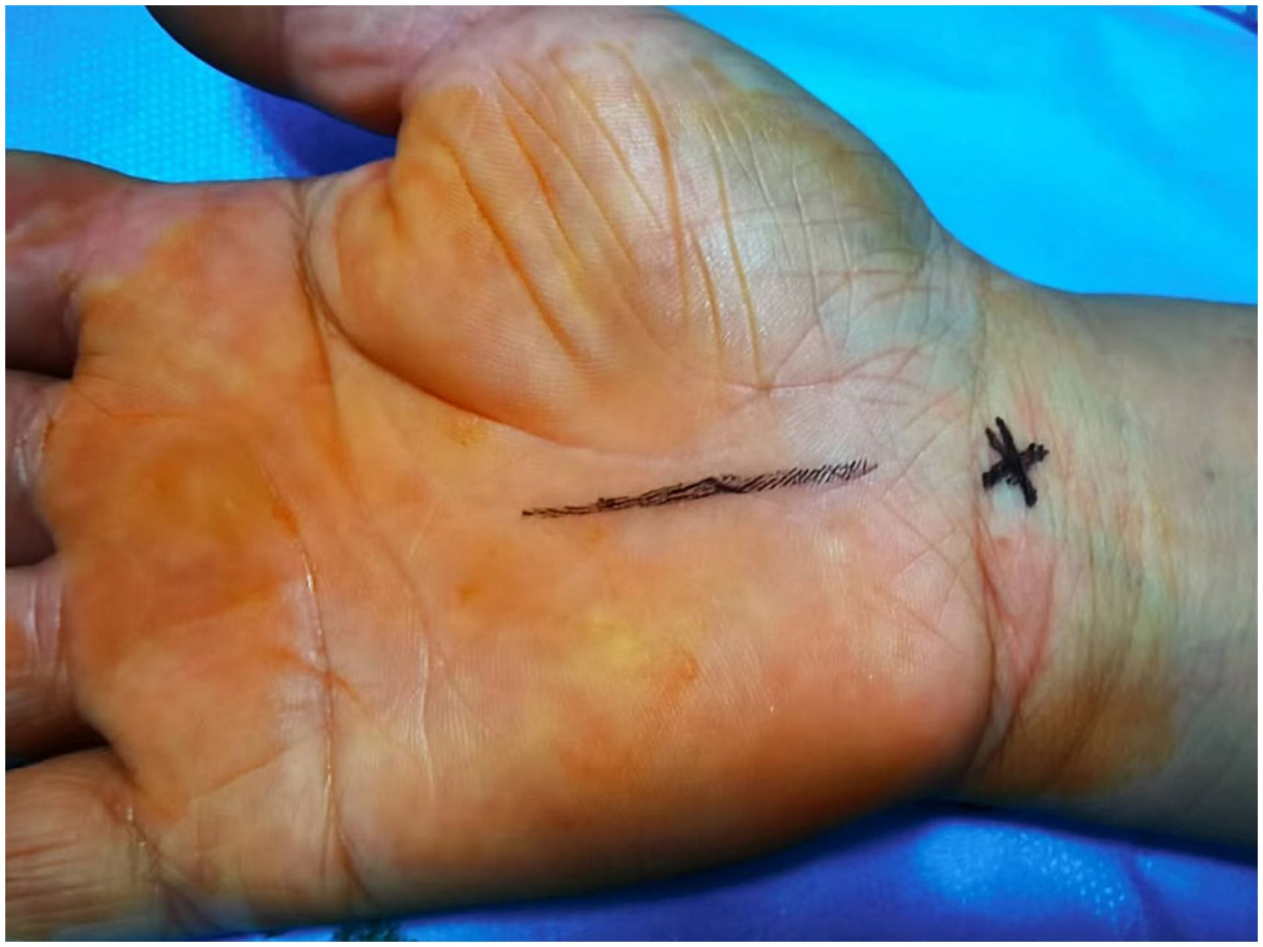
Puncture point located on the body surface. The x marks entry site. The vertical line marks the direction of advancing the needle knife.

**Figure 2:**
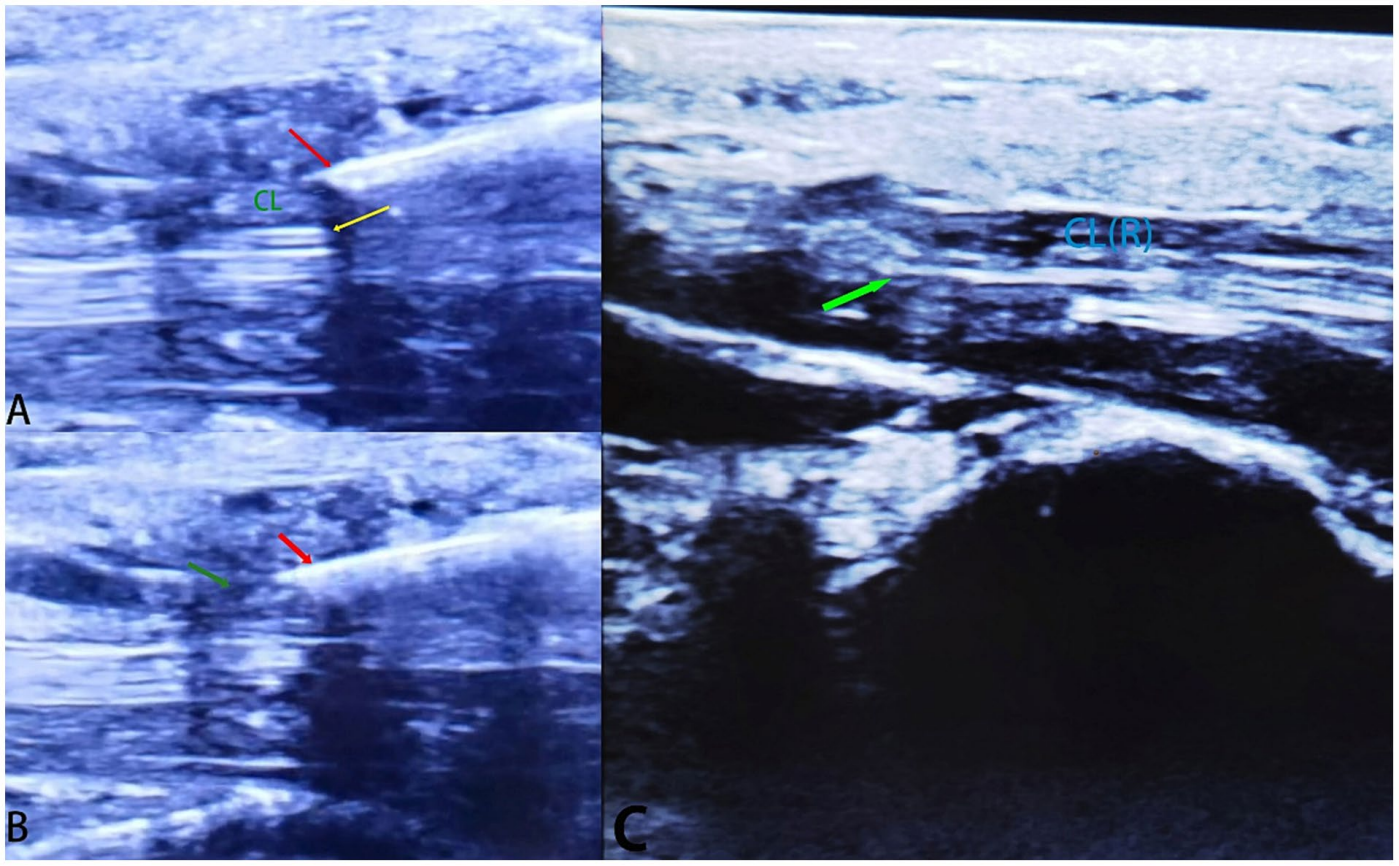
Ultrasound Images Demonstrating Median Nerve Compression and Carpal Ligament Release **(A)** Ultrasound image illustrating the location of median nerve compression before carpal ligament release. The compressed median nerve is indicated by the yellow arrow, and the carpal ligament is labeled as “CL.” Red arrow marks the needle knife. **(B)** The needle knife is slowly and carefully advanced (red arrow) to incise the carpal ligament, relieving pressure on the median nerve (MN). Green arrow marks the partly sectioned carpal ligament. **(C)** Ultrasound image showing the same area after carpal ligament release. The relieved median nerve is highlighted by the green arrow, and the incised carpal ligament is denoted as “CL(R).” The reduction in compression and enhanced nerve space can be observed.

The ultrasound-guided needle knife technique led to improved patient outcomes, reducing hand numbness and pain. Median nerve diameter increased after carpal ligament release, indicating successful decompression (see Figures 1, 2).

## 4. Discussion

Open release and medical management are two common approaches for treating CTS. Each approach has its advantages and disadvantages (19). Open release is a surgical procedure that involves cutting the transverse carpal ligament to relieve pressure on the median nerve. This technique has been shown to be effective in treating CTS, with high success rate and low complication rete (20). However, there are some disadvantages to open release, including a longer recovery time compared to minimally invasive techniques (21), potential scarring, and the risk of complications such as infection or nerve damage. Medical management for CTS typically involves conservative treatment such as wrist splinting, nonsteroidal anti-inflammatory drugs (NSAIDs), and corticosteroid injections. These treatments can provide temporary relief of symptoms and may be effective for mild cases for CTS. However, medical management may not provide long-term relief for more serve cases, and patients may eventually require surgery. Additionally, corticosteroid injections can have side effects, such as pain at the injection site and potential weaking of the tendons (22). The protocol introduces a novel and innovative approach—the ultrasound-guided needle knife technique—for the release of carpal ligament in the management of carpal tunnel syndrome (CTS). This technique presents a paradigm shift from traditional open surgeries, offering a range of distinct advantages that significantly enhance patient outcomes (23). In a clinical study using ultrasound-guided percutaneous release to treat stenosing flexor tenosynovitis with a needle knife, The results suggest that this technique is both safe and effective for the treatment of stenosing flexor tenosynovitis. The length and percentage of released A1 pulley were found to be longer with ultrasound guidance compared to non-ultrasound-guided techniques. The full release rate was highest in the ultrasound-guided group. However, it is important to note that the full release rate was still relatively low (13.6 to 31.4%) in all groups (24).

One of the primary advantages of the ultrasound-guided needle knife technique is its potential to expedite patient recovery. Compared to open surgical procedures, this minimally invasive approach involves a smaller incision, reduced tissue disruption, and decreased postoperative pain (24). As a result, patients may experience a quicker return to daily activities and work, mitigating the economic burden associated with extended recovery periods observed in conventional surgeries. The reduced trauma to surrounding tissues also contributes to the overall comfort of the patient during the postoperative phase. Another compelling benefit of the technique lies in the minimal to even no scarring it produces. The smaller incision and precision-guided nature of the needle knife approach result in esthetically pleasing outcomes that are particularly important in visible areas like the wrist (25). This can have positive psychological effects on patients, enhancing their body image and self-confidence during the recovery process. Furthermore, the real-time ultrasound guidance ensures accurate localization of the median nerve and precise targeting of the compressed ligament. This reduces the risk of inadvertent damage to adjacent structures, such as vessels or nerves, which is a concern in open surgeries (26). The technique’s accuracy contributes to a safer and more predictable procedure, further bolstering patient safety and positive outcomes. Several percutaneous ultrasound-guided approaches have been reported, including the “X” blade, needle release, hook knife, carpX, and thread carpal tunnel release (27). However, these methods still have certain limitations, such as the utilization of proprietary tools and a lack of larger clinical case studies. Among these techniques, the needle-knife technique has gained widespread usage and has been proven to be a cost-effective and efficient method (28).

The adoption of the ultrasound-guided needle knife technique not only offers immediate benefits to patients but also has wider implications for the field of hand surgery (29). The combination of ultrasound imaging and minimally invasive procedures is a promising avenue for advancing the treatment of various musculoskeletal and neurologic conditions. The technique’s success in CTS management highlights its potential to be adapted for other pathologies with similar anatomical constraints. Despite its numerous advantages, it is essential to acknowledge the potential limitations of the technique. The learning curve associated with ultrasound guidance and precise needle manipulation could impact procedural efficiency. As with any procedure, patient selection and thorough preoperative assessment remain pivotal for successful outcomes. While the ultrasound-guided needle knife technique offers significant advantages over traditional open surgeries, it is important to address potential challenges and consider its broader impact on clinical practice and research. One important aspect to consider is the role of operator expertise and training in the successful implementation of the technique. Ultrasound-guided procedures require a level of skill and familiarity with imaging technology. Ensuring that healthcare providers receive adequate training and hands-on experience is crucial to achieve consistent and optimal outcomes. As the technique gains popularity, specialized training programs and certification processes can contribute to its widespread adoption.

The long-term efficacy and durability of the results obtained from the ultrasound-guided needle knife technique warrant ongoing investigation. While short-term outcomes have shown improved patient comfort and function, longitudinal studies are necessary to assess the technique’s impact over extended periods. Monitoring patient outcomes, recurrence rates, and long-term nerve function will provide a comprehensive understanding of its sustainability. In the context of evolving healthcare practices, the economic implications of the ultrasound-guided needle knife technique should also be considered. While the technique may lead to faster recovery and reduced healthcare costs in the short term, comprehensive cost-effectiveness analyses are needed to assess its economic benefits in the long run. These analyses can inform healthcare decision-makers and policymakers when evaluating the integration of the technique into clinical practice. The successful adoption of the ultrasound-guided needle knife technique also highlights the importance of interdisciplinary collaboration. Close collaboration between radiologists, surgeons, and rehabilitation specialists is essential to optimize patient outcomes. Additionally, continuous collaboration between researchers and clinicians can drive further refinements of the technique based on real-world feedback and clinical insights. Furthermore, the technique’s successful application in carpal tunnel syndrome treatment raises intriguing possibilities for its adaptation in other clinical scenarios. Exploring its potential for other nerve compression syndromes, such as cubital tunnel syndrome or tarsal tunnel syndrome, could expand the scope of its clinical utility. This calls for multidisciplinary research efforts to explore its efficacy in various anatomical locations and patient populations.

## Data availability statement

The original contributions presented in the study are included in the article/Supplementary material, further inquiries can be directed to the corresponding author.

## Ethics statement

This study has been reviewed and approved by Ethical Review Board of Pujiang County Hospital of Traditional Chinese Medicine (2023C01). The written informed consent of all study participants has been obtained for this study. Written informed consent was obtained from the individual(s) for the publication of any potentially identifiable images or data included in this article.

## Author contributions

JS: Writing – original draft. XZ: Writing – original draft. QF: Writing – original draft. JW: Writing – review & editing, Formal analysis. SY: Writing – review & editing. AA: Writing – review & editing. YD: Writing – review & editing. HZ: Writing – review & editing. SA: Writing – review & editing. HL: Writing – review & editing.
